# Changing Mad2 Levels Affects Chromosome Segregation and Spindle Assembly Checkpoint Control in Female Mouse Meiosis I

**DOI:** 10.1371/journal.pone.0001165

**Published:** 2007-11-28

**Authors:** Théodora Niault, Khaled Hached, Rocío Sotillo, Peter K. Sorger, Bernard Maro, Robert Benezra, Katja Wassmann

**Affiliations:** 1 CNRS UMR7622 Biologie du Développement, Paris, France; 2 Université Pierre et Marie Curie (UPMC) Paris 6, Paris, France; 3 Inserm Avenir Team “Cell Division and Associated Checkpoints”, Paris, France; 4 Cancer Biology and Genetics Program, Memorial Sloan-Kettering Cancer Center, New York, New York, United States of America; 5 Department of Biology, Center for Cancer Research and Biological Engineering Division, Massachusetts Institute of Technology, Cambridge, Massachusetts, United States of America; 6 Department of Systems Biology, Harvard Medical School, Boston, Massachusetts, United States of America; 7 Department of Cell and Developmental Biology, Sackler School of Medicine, Tel Aviv University, Ramat Aviv, Israel; Ordway Research Institute, United States of America

## Abstract

The spindle assembly checkpoint (SAC) ensures correct separation of sister chromatids in somatic cells and provokes a cell cycle arrest in metaphase if one chromatid is not correctly attached to the bipolar spindle. Prolonged metaphase arrest due to overexpression of Mad2 has been shown to be deleterious to the ensuing anaphase, leading to the generation of aneuploidies and tumorigenesis. Additionally, some SAC components are essential for correct timing of prometaphase. In meiosis, we and others have shown previously that the Mad2-dependent SAC is functional during the first meiotic division in mouse oocytes. Expression of a dominant-negative form of Mad2 interferes with the SAC in metaphase I, and a knock-down approach using RNA interference accelerates anaphase onset in meiosis I. To prove unambigiously the importance of SAC control for mammalian female meiosis I we analyzed oocyte maturation in Mad2 heterozygote mice, and in oocytes overexpressing a GFP-tagged version of Mad2. In this study we show for the first time that loss of one Mad2 allele, as well as overexpression of Mad2 lead to chromosome missegregation events in meiosis I, and therefore the generation of aneuploid metaphase II oocytes. Furthermore, SAC control is impaired in *mad2+/−* oocytes, also leading to the generation of aneuploidies in meiosis I.

## Introduction

Chromosome missegregation events in mammalian female meiosis have severe consequences, leading to a drop in fertility due to the development of aneuploid embryos followed by spontaneous abortions. In humans, chromosome segregation errors in female meiosis I can lead to the development of trisomies, such as trisomy 21 [1]. The SAC has been shown to be required for the fidelity of chromosome segregation in mitosis, verifying the correct attachment of each pair of sister chromatids to the opposite poles of the bipolar spindle via their kinetochores [2]. The presence of a single erroneously attached kinetochore activates the SAC and leads to a metaphase arrest to permit repair of the attachment. Components of the SAC include the Mad (Mitotic Arrest Deficient) and Bub (Budding Uninhibited by Benomyl) proteins (Mad1-3, Bub1, BubR1, Bub3), as well as Mps1 (Monopolar Spindle 1), CenpE, and others [2]. Most SAC proteins are localized to unattached kinetochores in mitosis. Mad2 has a key role in that it directly inhibits the ubiquitination activity of the Anaphase Promoting Complex/Cyclosome (APC/C) in association with its activator Cdc20, and therefore anaphase onset. Once the SAC is inactivated, APC/C-Cdc20 ubiquitinates Securin and Cyclin B, targeting both proteins for degradation by the 26S proteasome. Securin is a protein inhibitor of a protease named Separase. Degradation of Securin leads to the activation of Separase which removes the cohesin complex holding sister chromatids together by cleaving one of its subunits, Scc1 [3]. Cdk1-Cyclin B complexes also inhibit Separase function and exit from mitosis, therefore Cyclin B needs to be degraded as well [4]. Hence inhibition of APC/C-Cdc20 by the SAC prevents sister chromatid separation in mitosis. In addition to its role at the metaphase-to-anaphase transition it has been shown that the SAC components Mad2 and BubR1 are implicated in the correct timing of prometaphase length. Knockdown of either protein leads to accelerated anaphase onset independent of their role in SAC control upon spindle disruption in metaphase, and independent of kinetochore attachment of both proteins [5].

In meiosis I, chromosome pairs each consisting of two sister chromatids are separated [6]. The kinetochores of two sister chromatids are oriented towards the same pole (monopolar attachment). This kind of attachment activates the SAC in mitosis, therefore the question at issue was whether the SAC can recognize a faulty attachment in meiosis I in mammalian oocytes. Chiasmata (sites of recombination) hold pairs of sister chromatids together throughout the first meiotic division, and therefore tension can be generated which should allow the silencing of the SAC [7]. On the other hand the existence of proper SAC control has been put into question by studies using XO female mice, which harbor one univalent X chromosome that cannot be properly attached and is segregated at random in meiosis I without causing a metaphase I arrest [8]. Recent studies have demonstrated that the SAC is present and detects attachment errors in female mouse meiosis I [9]–[12]. By RNA interference approaches and the use of a dominant negative Mad2 mutant it has been shown that the meiotic SAC depends on Mad2 as well [10]–[12]. Furthermore, injection of Mad2 morpholinos, or expression of dominant negative Mad2, Bub1, and BubR1 leads to an acceleration of meiosis I [10], [12].

In mitosis, it has been shown that loss of one Mad2 allele leads to the loss of 30% of Mad2 protein levels, and converts the chromosomal stable cell line Hct116 into a CIN (chromosomal instability phenotype) cell line [13]. *mad2+/−* mice are viable, but develop lung tumors with long latencies at elevated rates [13], whereas *mad2−/−* mice are embryonic lethal due to high chromosome loss [14].

By using mouse genetics we provide here the final proof that Mad2 is essential for correct chromosome segregation during normal first meiotic cell divisions in mouse oocytes, and not only upon treatment with spindle inhibitors such as nocodazole. Mammalian meiosis I is even more sensitive to loss of one allele of Mad2 than mitosis in the *mad2+/−* somatic cells studied so far. Furthermore, we show here for the first time that also Mad2 overexpression interferes with chromosome segregation in meiosis I.

## Results

**Figure 1:**
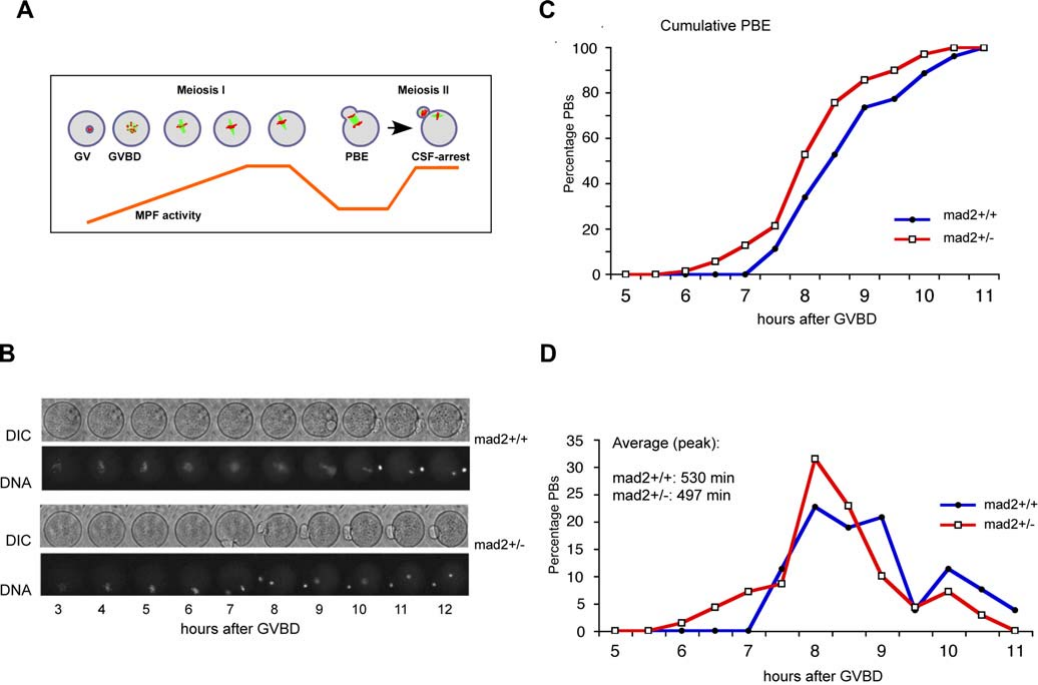
Anaphase onset is accelerated in *mad2+/−* mice. (A) Entry into the first meiotic division in mouse oocytes is induced by removing dbcAMP from the culture medium. Synchronized oocytes undergo GVBD around 1,5 hours after release, and extrude a PB around 8–9 hours after GVBD. MPF (M-phase promoting factor) activity increases from GVBD until metaphase I, drops when the first PB is extruded, and increases again as oocytes progress into meiosis II. (B) Time lapse video microscopy of oocytes with the indicated phenotype undergoing the first meiotic division. Chromosomes were labelled with Hoechst. Anaphase onset and PBE were observed. Only movies with at least 80% of oocytes extruding a PB at times comparable to control oocytes in the incubator without exposure to Hoechst excitation light were used. (C) Cumulative times of PBE and (D) distribution of PBE (same data set) in *mad2+/+* (n = 53) and *mad2+/−* (n = 70) oocytes. The peak time average of PBE in *mad2+/−* oocytes is significantly earlier (33 min) than in *mad2+/+* oocytes (497 min and 530 min respectively, p<0,01 with both the T and the U test, p value of the T test (2 tail, type2) = 0,00232, p value of the U test (2 tail) = 0,00572). The results of three independent experiments are shown.

To prove the importance of SAC control for female mouse meiosis I we examined the first meiotic division in oocytes derived from *mad2+/−* mice, compared to *mad2+/+* littermates. Denuded Germinal Vesicle (GV) stage oocytes arrested in prophase of meiosis I (after chiasmata have been formed and recombination has taken place) are collected from the ovaries of adult female mice. They can be induced to undergo meiosis I in culture in a completely synchronized manner. Cell division in meiosis I is asymmetric, and visible due to the extrusion of a small Polar Body (PB). Upon completion of meiosis I oocytes progress into metaphase of meiosis II where they remain arrested until fertilization occurs (Figure 1A). Control (littermates) and *mad2+/−* oocytes were analyzed by time lapse video microscopy to visualize chromosome movements (DNA was stained with Hoechst) and Polar Body Extrusion (PBE) with Phase Contrast (DIC) (Figure 1B). Photon excitation to visualize Hoechst is eventually toxic for oocytes, therefore we also followed meiotic maturation of untreated oocytes by determining the time of PBE through observation with a binocular microscope. Control oocytes of this strain extrude PBs 7.5 to 9.5 hrs after GVBD (Germinal Vesicle Breakdown, corresponds to Nuclear Envelope Breakdown in mitosis), with a peak time average at 8 h50 min (530 min), whereas PBE was significantly accelerated by 33 min on average (8 h17 min–497 min) in mad2+/− oocytes (Figure 1C, D). Meiosis I is 6,3% shorter in mad2+/− oocytes, which is comparable to the 10% decrease in the duration of mitosis observed after Mad2 RNAi [5]. We therefore conclude that Mad2 is important for correct timing of prometaphase in meiosis I, just like in mitosis [5].

**Figure 2:**
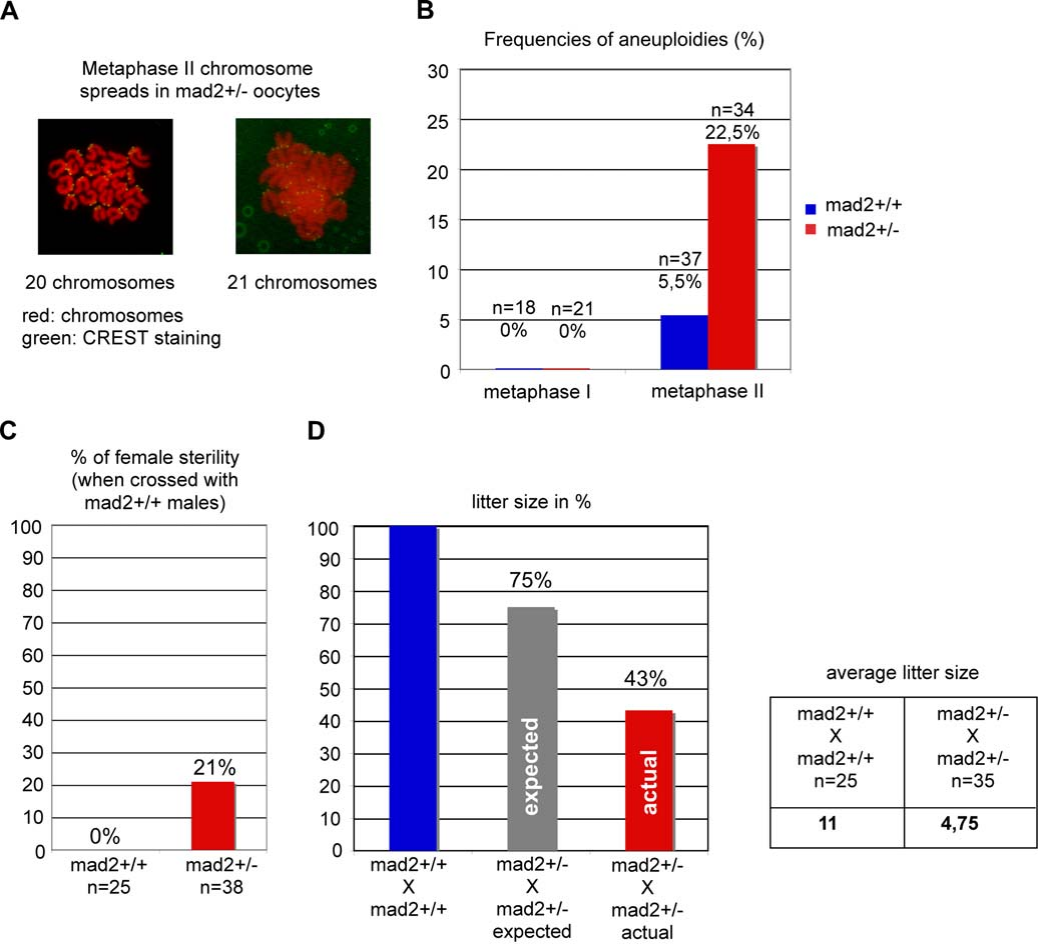
Loosing one allele of mad2 affects the fidelity of chromosome segregation in meiosis I and fertility. (A) Chromosome spreads were performed in metaphase I (6 hours after GVBD) and metaphase II (2 hours after PBE). Chromosomes are stained with propidium iodide (red), and kinetochores with CREST serum [30]. Shown are one euploid and one aneuploid metaphase II spread of *mad2+/−* oocytes. (B) Quantification of aneuploid oocytes in metaphase I and II harboring more or less than 20 chromosomes. n indicates the number of interpretable metaphase spreads obtained. 8 *mad2+/−* mice were analysed in four independent experiments. (C) 21% of female mad2+/− mice are sterile. (D) *mad2−/−* embryos are not viable and therefore the litter size is expected to be 25% lower in the heterozygote crosses. The average litter size from *mad2+/−* crosses is reduced to 43% compared to wild type crosses, and to 57,6% compared to the expected litter size.

The acceleration of anaphase onset in mad2+/− oocytes may not seem very striking at first sight as meiosis I is very long and takes around 9 hours from GVBD until PBE. It is important to take into account that the bipolar spindle self-assembles in mammalian oocytes without centrosomes in a very time consuming manner around condensed chromosomes [15], [16]. Stable microtubule-kinetochore interactions are established only 8 hours after GVBD [15], therefore activating the APC/C too early may have severe consequences because oocytes may still not have had enough time to establish stable microtubule-kinetochore interactions. We wanted to know whether loss of one allele of Mad2 leads to increased chromosome gain or loss in meiosis I. Chromosome spreads of oocytes before and after they had undergone the first meiotic division in culture were analyzed. The centromere region was stained with CREST serum for easier interpretation of the spreads (Figure 2A). Certain studies have put into question the in vitro culture of denuded Germinal Vesicle (GV) stage oocytes which can lead to meiotic delays and aneuploidies when not performed properly. We show here that control oocytes are euploid before and after meiosis I under our experimental conditions, demonstrating that our culture conditions do not induce aneuploidies per se (Figure 2B). *mad2+/−* oocytes harbor the correct number of bivalents before metaphase to anaphase transition in meiosis I, meaning that they did not accumulate aneuploidies at significant rates during the premeiotic divisions. Importantly, *mad2+/−* oocytes are aneuploid at elevated rates (22,5%) in metaphase of meiosis II due to chromosome missegregation in meiosis I (Figure 2B). The presence of more or less than 20 univalents was observed in aneuploid oocytes (Figure 2A). It is important to note that mitotic Hct-116 *mad2+/−* cells become aneuploid at 32 to 50% after 25 generations [13], whereas we observe aneuploidies at more than 20% after only one meiosis I division in mouse oocytes. We demonstrate here for the first time that loss of one allele of a SAC gene leads to a significant increase in chromosome loss specifically during the first meiotic division.

Elevated chromosome missegregation levels in meiosis I are expected to be correlated with a drop in fertility in *mad2+/−* female mice. Indeed, 21% of *mad2+/−* female mice are sterile (Figure 2C), whereas no effect on male fertility was detected upon loss of one Mad2 allele (data not shown). Furthermore, litter size is reduced by more than half in *mad2+/−* X *mad2+/−* crosses. An expected loss of 25% has to be taken into account, as *mad2−/−* embryos are not viable [14], which leaves the net loss in litter size at 42,4% compared to the expected size (Figure 2D). We conclude that fertility is affected in *mad2+/−* mice, and even though we do not have the final proof that this drop in fertility is due to chromosome missegregation events in meiosis I, it is attractive to speculate that increased aneuploidies in *mad2+/−* oocytes and the observed drop in fertility are cause and consequence of the same deficiencies in meiosis I.

**Figure 3:**
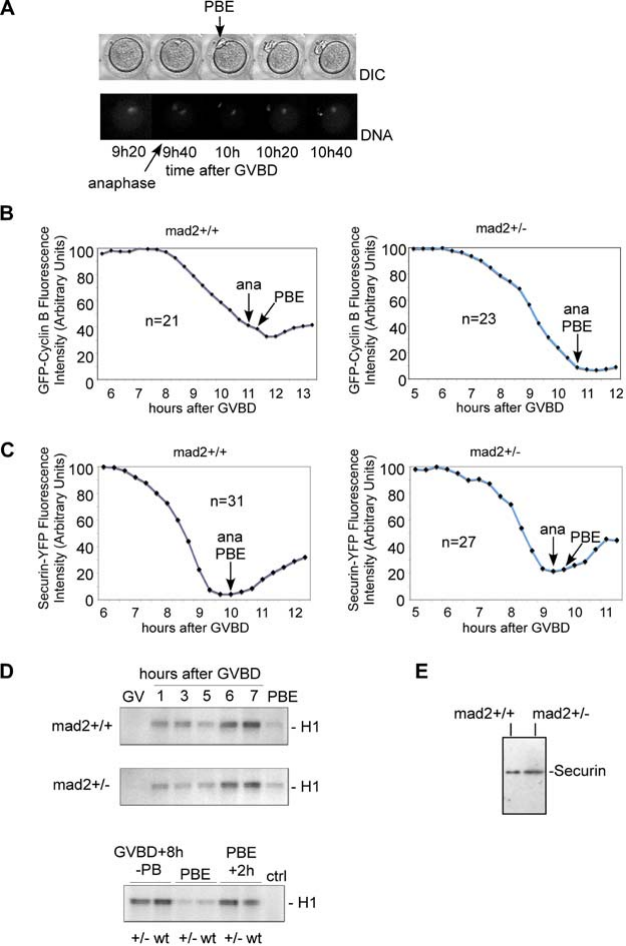
Cyclin B and Securin are degraded before PBE in *mad2+/−* oocytes. (A) Time lapse video microscopy to establish the time of anaphase onset and PBE. Anaphase onset can sometimes be observed in the timepoint before PBE. (B) Quantitation of GFP-Cyclin B in *mad2+/+* and *mad2+/−* oocytes relative to anaphase onset and PBE. Live video analysis shows that GFP-Cyclin B (quantifications of the fluorescence signal are shown as a graph, arbitrary units set to maximal levels of 100) reaches its lowest levels before PBE. Timepoints were taken every 20 minutes, chromosomes were labelled with Hoechst. A representative oocyte is shown and the number of successfully analyzed oocytes is indicated. No differences between anaphase onset, PBE and GFP-Cyclin B degradation were detected between control and *mad2+/−* oocytes. (C) Quantifications of Securin-YFP fluorescence signal intensities in *mad2+/+* and *mad2+/−* oocytes relative to anaphase onset and PBE, as described in (B). No differences between anaphase onset, PBE and Securin-YFP degradation were detected. (D) Kinase assays to assess MPF activity during meiosis I in *mad2+/+* and *mad2+/−* oocytes. Histone H1 (H1) was used as a substrate. Control (ctrl): GVBD+8 h (-PB) without substrate addition. (E) Endogenous Securin levels in *mad2+/+* and *mad2+/−* oocytes 3 hours after GVBD. 20 oocytes each were used.

We set out to see whether known hallmarks of cell cycle regulation occur at the right time in *mad2+/−* oocytes. We established a time lapse video microscopy assay to determine when anaphase onset and PBE take place relative to Cyclin B and Securin degradation in female mouse meiosis I. GFP-Cyclin B and Securin-YFP expressing mRNAs were injected into GV stage oocytes, and their degradation was followed by quantification of the fluorescence signal. As shown previously, overexpression of Cyclin B delays PBE in a dose dependent manner [17]. The first acquisition was taken 40 minutes earlier in *mad2+/−* oocytes to account for earlier PBE. Chromosomes were stained with Hoechst, and PBE was observed by phase contrast (DIC) (Figure 3A). In control oocytes, anaphase onset occurs just before GFP-Cyclin B protein levels are the lowest (Figure 3B), but always after YFP-Securin has reached its lowest levels (Figure 3C). All *mad2+/−* oocytes show the same pattern of GFP-Cyclin B and Securin-YFP degradation relative to anaphase onset and PBE (Figure 3B and C). Depending on the amount of Securin-YFP or GFP-Cyclin B injected, anaphase onset and PBE are delayed in both, control and *mad2+/−* oocytes. It has to be taken into account that the time points are only every 20 minutes (more frequent acquisitions to visualize both, chromosomes and Securin-YFP or GFP-Cyclin B were lethal for the oocytes due to the toxicity of the excitation light), therefore small differences in the degradation profiles may not have been detected. Nevertheless, we think this is unlikely due to the large numbers of oocytes observed.

In control oocytes, MPF activity increases from GVBD until metaphase I, and drops sharply upon PBE [18]. In *mad2+/−* oocytes, endogenous MPF activity disappears upon PBE, as in the control (Figure 3D). We decided to explore the possibility that lower Securin levels in *mad2+/−* oocytes are responsible for accelerated anaphase onset. No differences in Securin levels between control and *mad2+/−* oocytes were observed (Figure 3E), therefore we cannot explain the accelerated anaphase I onset in *mad2+/−* oocytes with a detectable increase in Securin degradation. We conclude that loss of one Mad2 allele leads to a general acceleration, but not de-regulation of the cell cycle in prometaphase and metaphase of meiosis I, and this acceleration may not leave enough time for proper attachment of kinetochores to the bipolar spindle in meiosis I and may consequently lead to the generation of aneuploidies.

**Figure 4:**
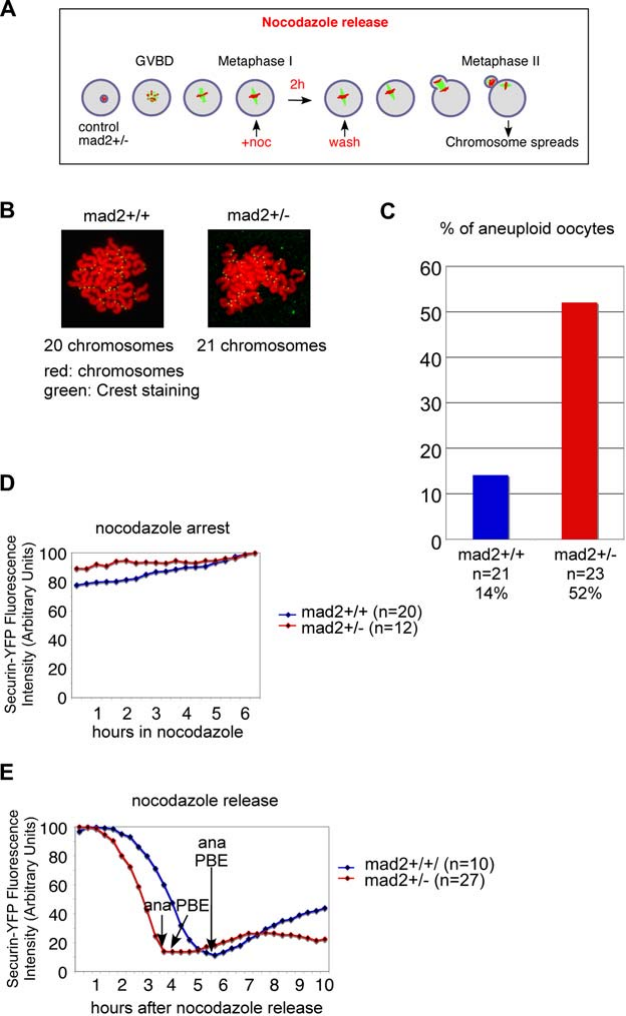
SAC control is impaired in *mad2+/−* oocytes. (A) Schematic outline of the experimental procedure. Nocodazole was used at a final concentration of 200 nM. (B) Chromosome spreads in metaphase II. Shown are one euploid *mad2+/+* oocyte and one aneuploid *mad2+/−* oocyte harboring 21 chromosomes. Chromosomes are stained with propidium iodide (red), and kinetochores with CREST serum [30]. (C) Percentage of aneuploid oocytes, as determined by metaphase II spreads and kinetochore staining. 4 independent experiments using 4 mice of each genotype were performed. (D, E) Time lapse video microscopy of oocytes expressing Securin-YFP as described in Figure 3. The graphs show the fluorescence measurements of Securin-YFP at the indicated times after GVBD, and the times of anaphase onset and PBE. Timepoints were taken every 20 minutes, chromosomes were labelled with Hoechst. A representative oocyte is shown and the number of successfully analyzed oocytes is indicated. (D) Oocytes treated with nocodazole. (E) Oocytes released from a 2 hour nocodazole arrest as in (A–C).

Loss of one allele of Mad2 is expected to affect SAC control, and this may lead to a premature activation of the APC/C and chromosome missegregations. Therefore, we determined whether SAC control is still functional in *mad2+/−* oocytes. As we have shown previously, mouse oocytes maintain a metaphase I arrest upon treatment with low doses of nocodazole for several hours [11]. But similar to primary tissue culture cells in mitosis, around 40 percent ultimately escape a prolonged nocodazole-induced metaphase I arrest without having correctly attached their chromosomes [11]. To properly compare SAC function in mad2+/+ and mad2+/− oocytes (we did not expect a complete loss of SAC control as one allele of Mad2 is still present) without being biased by potential differences in adaptation to nocodazole treatment we performed a nocodazole arrest and release experiment (Figure 4A). Oocytes were treated with low doses of nocodazole in metaphase I for 2 hours, before release into medium without nocodazole. Metaphase II chromosome spreads with centromere staining of oocytes that extruded a PB showed that control oocytes were able to transiently arrest in metaphase I upon treatment with 200 nM nocodzole and to segregate chromosomes correctly upon nocodazole release (Figure 4B and C). Treatment with higher doses of nocodazole leads to increased missegregation events upon release also in control oocytes in this strain background (data not shown), therefore 200 nM nocodazole were used to assess the capacity of *mad2+/−* oocytes to respond correctly to spindle damage. We show here that *mad2+/−* oocytes missegregate their chromosomes at elevated rates after release from nocodazole arrest, indicating that loss of one allele of Mad2 severly perturbs SAC control (Figure 4B and C). As before, loss or gain of univalents, and the presence of bivalents were observed. Nevertheless, GFP-Cyclin B and Securin-YFP are stabilized in nocodazole treated *mad2+/−* oocytes (Figure 4D), and degraded on time before anaphase onset (Figure 4E). We conclude that the SAC is prematurely inactivated in *mad2+/−* oocytes, resulting in degradation of APC/C substrates and anaphase onset with chromosome losses. Taken together our results show for the first time a direct connection between SAC loss and incorrect chromosome segregation by mouse genetics in mammalian female meiosis I.

**Figure 5:**
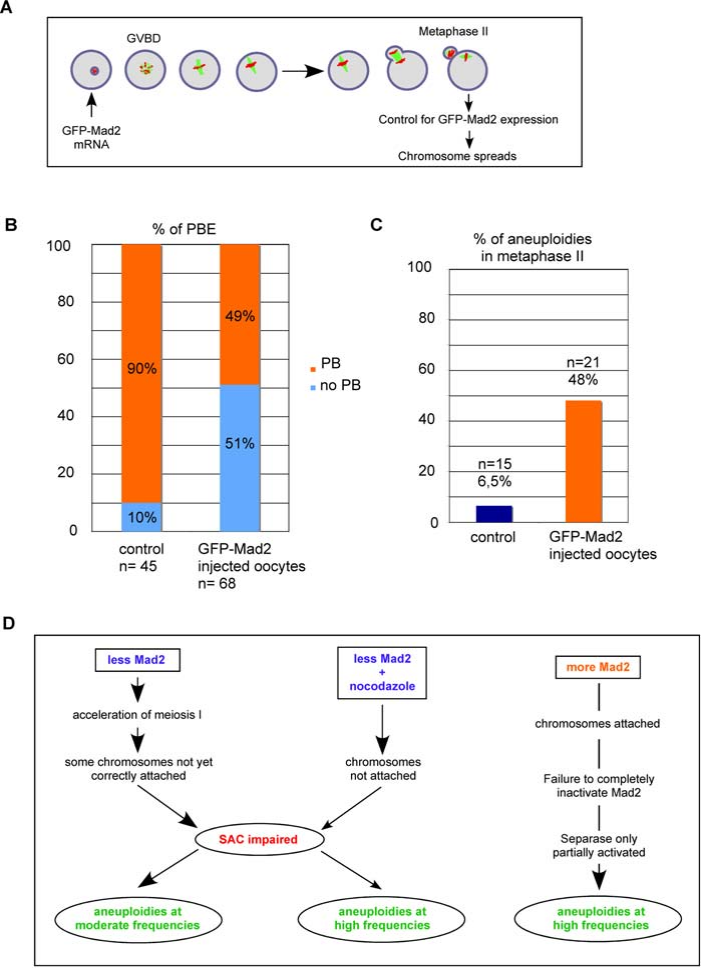
Overexpression of Mad2 leads to chromosome missegregations in meiosis I. (A) Schematic outline of the experimental procedure. Oocytes arrested in GV stage are injected with mRNA encoding GFP-tagged Mad2, and released to undergo meiotic maturation. Oocytes that extrude a PB are examined for GFP-Mad2 expression by fluorecence microscopy, and those expressing detectable levels of Mad2 are used for chromosome spreads. The experiment was repeated 4 times. (B) Percentage of oocytes bypassing the metaphase I arrest with moderate GFP-Mad2 expression. (C) Percentage of aneuploidies in metaphase II (gain or loss of one or more univalent chromosomes) of oocytes injected with injection buffer (control), or GFP-Mad2 mRNA (same as in (B)). (D) Model to explain the observed phenotypes after changing Mad2 levels. See text for details.

Overexpression of Mad2 activates the SAC and leads to an arrest in metaphase [19], [20]. In Mouse Embryonic Fibroblasts (MEFs), expression of exogenous Mad2 at moderate levels also delays mitosis and has been shown to cause chromosomal instability as cells attempt to bypass the metaphase arrest, probably without completely removing the cohesin complex holding the sister chromatids together [21], [22]. In light of the severe effects observed in mitotic primary tissue culture cells, we decided to address whether moderate expression of Mad2 has the same effects in meiosis I in mouse oocytes. GFP-tagged Mad2 which we have shown to be functional to activate the SAC in a previous study [11] was overexpressed at moderate levels in GV stage oocytes. Oocytes were induced to undergo meiotic maturation, and those which extruded a PB (51%) were analyzed by metaphase II spreads together with centromere staining as above (Figure 5A, and B). Expression of GFP-Mad2 was controlled by fluorescence microscopy. 48% of GFP-Mad2 expressing oocytes in metaphase II were aneuploid, confirming that as in mitosis overexpression of Mad2 in meiosis I leads to a CIN phenotype (Figure 5C).

## Discussion

Our model in Figure 5D illustrates how we explain the generation of aneuploidies in meiosis I upon changing Mad2 levels in mouse oocytes: Reducing Mad2 levels leads to an acceleration of meiosis I, not leaving enough time for proper attachment of the bipolar spindle to functional kinetochores in metaphase I. SAC function is affected, and therefore oocytes with unattached kinetochores will eventually progress into meiosis II. Consistent with our observation a correlation between the concentration of transcripts of SAC components, and missegregation events in human oocytes has been described: the authors show that there seems to be an age-dependent decrease in Mad2 and Bub1 transcripts that may potentially underlie the increased incidence of aneuploidies in older women [23].

Upon nocodazole arrest and release, the effects of a malfunctioning SAC become more severe, because more unattached kinetochores need to be properly attached and have to be recognized by the SAC. Overexpression of Mad2 on the other hand leads to SAC activation, but as all kinetochores are attached, mechanisms to inactivate the SAC will be activated at the same time, too. It is attractive to speculate that mechanisms required to inactivate Mad2 are too weak to overcome the entire pool of active Mad2 molecules in the overexpression experiment, but still strong enough to inactivate a fraction of Mad2, therefore allowing partial activation of the APC/C. Separase may only be partially activated, either due to a failure to completely degrade Securin, or a failure to inactivate MPF which may keep a fraction of Separase inactivated by inhibitory Cdk1 binding [24], [25]. As a consequence, not all Cohesin is removed from chromosomes and this leads to the missegregation events observed. Indeed, the same phenotype, namely aneuploidies in metaphase II mouse oocytes, has been observed upon injection of a peptide inhibitor of Separase that probably only incompletely inhibits Separase [26], whereas complete loss of Separase function in mouse oocytes blocks all separation of bivalent chromosomes [27].

In summary, correct metaphase-to-anaphase transition depends on a fine balance between not enough, or too much SAC activity, both having severe consequences on the fidelity of chromosome segregation both in mitosis and meiosis. We show here that the female first meiotic division is much more sensitive to loss of one Mad2 allele than what has been observed in mitotic cells [13]. Our results help to understand how missegregation events in meiosis I can ultimately lead to the generation of trisomies or spontaneous abortions in humans. Future studies using oocyte specific gene invalidation of essential SAC genes will be required to further address the role of the SAC for correct chromosome segregation in meiosis I in mammalian oocytes.

## Materials and Methods

### Mouse oocyte culture

The *mad2* mouse strain has a *C57BL/6J* and *129/Sv* mixed background. Genotyping of *mad2+/+* and *mad2+/−* mice was done as described [14]. For microinjection experiments (Figure 5) OF1 mice (Charles River, France) were used. Mouse oocytes were harvested from 9 to 16 week old mice and cultured in M2 medium (Sigma) as described [11]. Oocytes were maintained in GV stage by the addition of dibutyryl cyclic Amp (dbcAmp) at 100 µg, and released into M2 medium without dbcAmp to undergo meiotic maturation. Oocytes were re-synchronized at GVBD.

### Plasmids, synthesis and microinjection of capped mRNAs

Mouse Securin cDNA was amplified with PfuTurbo DNA polymerase (Stratagene) according to the manufacturer's protocol from pRN3Myc2securin [26] with the following primers: 5′-CCCTTCGAATTCGCCACCATGGCTACTCTTATCTTT-3′ and 5′-GAAGTAAGGATCCC GAATATCTGCATCGTAACAGCC-3′, which introduces an EcoR1 site at the N-terminus, and a BamH1 site at the C-terminus. The EcoR1-BamH1 fragment was cloned into pEYFP-N1 (BD Biosciences Clontech) to generate a C-terminal YFP-tagged version of mouse Securin. Securin-YFP was cloned into pRN3[28] as an EcoR1-Not1 fragment for capped mRNA production with the mMessage mMachine T3 kit (Ambion). GV-stage oocytes were injected with GFP-Mad2 [11], CyclinB-GFP [17], or Securin-YFP encoding capped mRNAs, or injection buffer (control) with Eppendorf micromanipulators and a FemtoJet microinjector (Eppendorf) using continuous flow settings. Oocytes were released into M2 medium within 1 to 3 hours after injection.

### Time-lapse video microscopy of live oocytes

Time lapse video microscopy was performed on a Leica DM IRBE microscope equipped with a Micromax 1300 YHS CCD camera (Princeton Instruments). MetaMorph software (Universal Imaging) was used for the acquisition of DIC and fluorescence images. Oocytes were incubated 1–2 hours in Hoechst 33342 (Sigma, 2 ng/ml) containing M2 medium prior to time lapse microscopy. The excitation light of a 100 W mercury lamp was decreased to 10% with a neutral density filter, and acquisitions were taken every 20 minutes with constant exposure times throughout the time lapse. YFP and GFP signal intensities were calculated as the sum of pixels in a defined region against mean background levels with Image J 1.37v software (NIH). GFP-Mad2 expression was controlled by doing one acquisition to visualize the GFP of single oocytes with constant settings.

### Kinase assays, western blots

Kinase assays and western blots were performed as described previously [11]. Mouse anti-Securin antibody (Abcam, 3305), peroxidase coupled secondary anti-mouse antibody (Immuno Research) and the Immobilon Western Chemiluminescent HRP substrate (Millipore) were used for visualization. For kinase assays, 10 oocytes were taken for each reaction at the indicated time points. Oocytes before and after PBE were manually removed under the microscope.

### Statistical analysis

In Figure 1, anaphase onset times (grouped by intervals of 30 min) for 50 or more oocytes were plotted as a result of 3 independent experiments. PBE time corresponds to the time when oocytes start extruding a visible PB. The peak time corresponds to the average PBE time. Significance was determined by the one-tailed Wilcoxon Rank sum test (n(mad2+/−) = 70, n(mad2+/+) = 53, U = 2387,5) and the un-paired Student's T test, both giving a p<0,01, which is considered as highly significant (p value of the T test (2 tail, type2) = 0,00232, p value of the U test (2 tail) = 0,00572.).

### Chromosome spreads, CREST staining, and image acquisitions

For chromosome spreads, the zona pellucida was removed with tyrode's solution (Sigma). Spreads were done as described[29]. For CREST staining, spreads were washed with PBS, blocked for 30 minutes with 1,5% BSA in PBS, and incubated with anti-CREST serum (HCT-100, Immunovision) at a dilution of 1∶50 in the blocking solution for several hours. After three washes with PBS spreads were incubated with anti-human Alexa 488 (Sigma) at a dilution of 1∶200 in the blocking solution for 1 hour. Chromosomes were washed three times with PBS, stained with propidium iodide (1 µg/ml in PBS) for 10 minutes, washed again and covered with mounting medium (AF-1, Citifluor) and a cover slip.

### Animal breeding

Mouse breeding and genotyping were done as described [14].
